# Bio-banding in junior soccer players: a pilot study

**DOI:** 10.1186/s13104-020-05083-5

**Published:** 2020-05-12

**Authors:** Michael Romann, Dennis Lüdin, Dennis-Peter Born

**Affiliations:** Department for Elite Sport, Swiss Federal Institute of Sport Magglingen, Alpenstrasse 18, 2532 Magglingen, Switzerland

**Keywords:** Talent development, Maturation, Puberty, Youth sport, Youth competition

## Abstract

**Objective:**

Bio-banding (BB) has been introduced to account for varying maturity and to improve the talent development of junior soccer players. To date, research that investigated the physiological and technical effects of BB is sparse. Therefore, the aim of the study was to compare effects of BB with CA on selected technical and tactical parameters in U13 and U14 soccer players.

**Results:**

BB significantly increased the number of duels (*p *= 0.024) and set pieces (*p *= 0.025) compared to chronological age. The mean time of ball possession per action was reduced (*p *= 0.021) and the rate of successful passes was lower with BB (*p *= 0.001). Meanwhile, the total number of passes was unaffected (*p *= 0.796), and there was a trend towards a lower difference in ball possession between BB teams (*p *= 0.058). In addition, BB reduced the distances covered while jogging (*p *= 0.001), running (*p *= 0.038) and high-speed running (*p *= 0.035). With BB, an increased number of duels, unsuccessful passes and set pieces resulted in a quicker change of match play situations between teams. While physical demand was reduced, BB seems to result in a more technically and tactically challenging game. Benefits in long-term player development, however, require further investigation.

## Introduction

The biological age (BA) of soccer players shows a large variation during puberty [1]. Early maturing players tend to dominate match play due to superior physical abilities, i.e. running speed, body height and mass [1], and they are promoted more likely in the talent selection process. In U16 academy soccer players, for instance, 50% were early and only 1.9% late maturing individuals [2]. However, late maturing players catch up with their biological development and might even overtake early developed counterparts with superior technical [3] and psychological skills [4] when not being deselected and lost at an earlier stage of talent selection [5, 6]. To account for varying maturation, junior soccer players might be grouped by BA rather than chronological age (CA) for training and competition [1, 7]. So-called bio-banding (BB) might help to overcome variations in maturity and might benefit both late and early maturing players. During BB matches, late maturing players perceived a reduced injury risk [2, 4], despite being involved in more tackles [8]. In addition, they had better chances to demonstrate their technical and tactical abilities [2, 4]. For early maturing players, BB provided more technical challenges that might aid in the holistic development of soccer talents [2, 4]. In previous studies, players were bio-banded over a wide range of age groups (11–15 years [8] and 9–15 years [2]), which might be practical for large clubs and soccer academies. Smaller clubs, however, do not have access to such a large number of players. In addition, in most countries, soccer teams are composed over a 2-year age span. Therefore, as recommended previously [1, 7], the aim of the study was to compare effects of BB with CA on selected technical and tactical parameters in U13 and U14 soccer players.

## Main text

### Methods

#### Participants

The present pilot study assessed BB based on practical considerations in a paired crossover design. In total, n = 33 U13 soccer players (32 male, 1 female; body height: 151 ± 8 cm, body mass: 38 ± 6 kg, age: 12.2 ± 0.3 years, maturity offset: − 1.6 ± 0.8 years) and n = 29 U14 soccer players (28 male, 1 female, 157 ± 6 cm, 45 ± 5 kg, 13.2 ± 0.4 years, − 0.8 ± 0.6 years) were evaluated. All players were recruited from two elite soccer clubs participating in the Swiss talent development programme [9]. The programme represents the national top 2000 U13 and top 1200 U14 players. All players from the present study participated for at least 4.3 ± 0.7 years in structured and regular soccer practice and match play. After ethical approval (Swiss Federal Institute of Sport; Nr. 2018/057), all participants and their legal guardians signed written informed consent to participate. The study was conducted in accordance with the guidelines of the Declaration of Helsinki.

### Procedures

#### Bio-banding

Maturity offset (MO), age at peak height velocity (APHV) and percentage of adult height were assessed by measuring body weight, standing height and sitting height using a body scale (Seca 876, Seca, Hamburg, Germany) and a stadiometer (Seca 217, Seca, Hamburg, Germany; Mirwald, 2002; Sherar, 2005). Subsequently, all male players were ranked based on their MO. The ranking was split at its median and the biologically younger half assigned to team BA1 (MO: − 2.5 to − 1.2 years) and biological older half to BA2 (MO: − 1.2 to 0.0 years). Both female players were on-time maturing and therefore stayed with their team. With respect to practical considerations and previous recommendations [1, 7], team coaches evaluated each player’s technique, game intelligence, personality and playing speed (TIPS) [10–12]. TIPS is the talent identification instrument invented by Ajax Amsterdam. The model has been mirrored by several clubs of the English Premier League and the Swiss Football Association. Based on the coaches’ evaluation, two players from BA1 were assigned to BA2 due to their superior technical and tactical abilities. Two players from BA2 still had to catch up with their technical and tactical skills and were assigned to BA1.

#### Match play

After a two-month familiarisation period with BB training sessions and matches, eight 20-min matches were carried out on two natural grass pitches (size: 55 × 58 m). The first and fourth 20-min matches were played with BB, while the second and third 20-min matches were played with conventional CA groups (U13 and U14). The matches were video-captured ball-oriented (focused on the ball) for a subsequent analysis of technical and tactical parameters. The camera was positioned near the middle line of the pitch at a height of 3.5 meters above the ground. Video data were coded and analysed using DartFish Note (Dartfish, Fribourg, Switzerland). Technical and tactical parameters were defined beforehand by an expert group of the Swiss Football Association. These parameters were: difference in ball possession between teams (% p.), mean time of ball possession per action (s), changes in ball possession (n), shots (n), successful goal attempts (%), passes (n) (successful% vs unsuccessful%), duels (n), fouls (n) and set pieces (n) which were subdivided by goal kicks, throw-ins, free kicks and penalty kicks. Physical parameters were measured using global positioning data (FieldWiz, Sensitech AG, Goldach, Switzerland) collected with 18-Hz. Because some players were substituted between matches, 49 of 72 possible paired subjects playing both BB and CA matches were used for the analysis. Based on previous recommendations, running distances were analysed for walking, jogging, running, high-speed running and sprinting at 0–5.8 km/h, 5.8–11.5 km/h, 11.5–15.8 km/h, 15.8–20.0 km/h and > 20.0 km/h, respectively [13].

#### Statistical analysis

All data are presented as mean ± standard deviation (SD). All matches were divided into 5-min intervals, from which mean values were used to compare differences between BB and CA using a t-test. Wilcoxon’s signed-rank test was applied for all non-normally distributed data (Shapiro–Wilk test). Cohen’s *d* effect sizes were calculated [14] and regarded as small (< 0.2), medium (< 0.6) and large (< 1.2) [15]. Statistically significant differences were analysed with SPSS 25.0 (IBM Corp., Armonk, NY, USA) and an alpha level of 0.05.

### Results

#### Anthropometrical and physical parameters

**Fig. 1:**
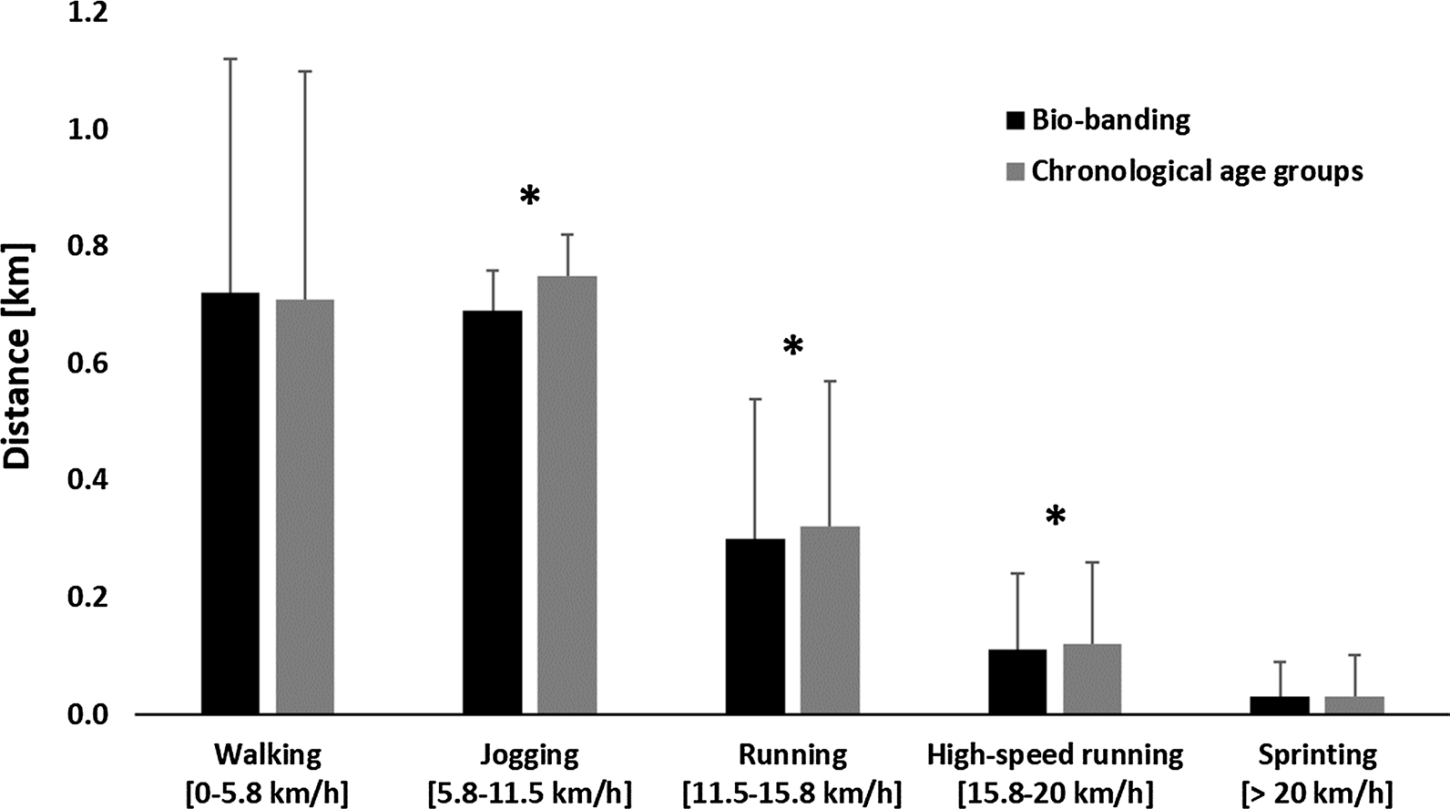
Physical parameters for bio-banded compared to conventional match play. *Significant difference between groups

MO (− 1.69 ± 0.74 and − 0.74 ± 0.59 years; *p *< 0.001, *d *= 1.42) and percentage of adult height (83.6 ± 1.6% and 87.8 ± 2.8%; *p *= 0.008, *d *= 1.24) were significantly different between the BB teams BA1 and BA2, respectively. APHV (13.96 ± 0.59 and 13.79 ± 0.40 years; *p *= 0.197, *d *= 0.31) were similar in both groups. Physical data are presented in Fig. 1. BB reduced the total running distance (1.83 ± 0.40 and 1.94 ± 0.39 km; *p *< 0.001, *d *= 0.26), as well as distances covered jogging (0.69 ± 0.24 and 0.75 ± 0.25 km; *p *< 0.001, *d *= 0.25), running (0.30 ± 0.13 and 0.32 ± 0.14 km; *p *= 0.038, *d *= 0.22) and high-speed running (0.11 ± 0.06 and 0.12 ± 0.07 km; *p *= 0.035, *d *= 0.25) compared to CA, respectively. Distances for walking (0.72 ± 0.07 and 0.71 ± 0.07 km; *p *= 0.714, *d *= 0.05), sprinting (0.03 ± 0.03 and 0.03 ± 0.03 km; *p *= 0.436, *d *= 0.13), maximal running velocity (22.19 ± 3.07 and 21.98 ± 2.57 km/h, *p *= 0.567, *d *= 0.07) and maximal accelerations (4.11 ± 0.40 and 4.08 ± 0.38 m/s^2^, *p *= 0.700, *d *= 0.06) were similar between BB and CA.

#### Technical and tactical parameters

Compared to the CA match play, BB significantly increased the number of duels (p = 0.024; *d *= 0.89) and set pieces (p = 0.025; *d *= 1.00), while the mean time of ball possession per action (p = 0.021; *d *= 0.62) was reduced. The rate of successful passes (p < 0.001; *d *= 1.24) was lower and the rate of unsuccessful passes (p < 0.001; *d *= 1.06) was higher with BB, but the number of passes remained unaffected. A trend towards a lower difference in ball possession between bio-banded teams was evident (*p *= 0.058; *d *= 0.69). The remaining technical and tactical parameters were similar between the BB and CA conditions (Table 1).

**Table 1:** Technical and tactical parameters for bio-banded compared to conventional match play based on chronological age (CA) groups

Parameter	BB	CA	*p*-value	95% CI	Cohen’s *d*
Duels [*n*]	6.8 ± 3.4	4.4 ± 1.7	0.02	[0.4, 4.4]	0.89
Difference in ball possession between teams [% p.]	6.0 ± 16.0	18.8 ± 20.6	0.06	[− 26.1, 0.5]	0.69
Mean time of ball possession per action [s]	10.7 ± 2.7	12.4 ± 2.7	0.02	[− 3.0, − 0.3]	0.62
Changes in ball possession [*n*]	30.4 ± 8.3	26.3 ± 6.6	0.08	[− 0.5, 8.8]	0.55
Fouls [*n*]	1.1 ± 0.9	1.3 ± 1.3	0.57	[− 0.9, 0.5]	0.17
Set pieces [*n*]	10.3 ± 2.9	7.5 ± 2.6	0.03	[0.4, 5.1]	1.00
Shots [*n*]	3.4 ± 2.0	3.8 ± 1.8	0.53	[− 1.6, 0.9]	0.20
Successful goal attempts [%]	30.3 ± 38.6	25.6 ± 23.1	0.76	[− 14.6, 23.9]	0.15
Passes [*n*]	41.4 ± 7.5	40.6 ± 10.9	0.80	[− 6.2, 8.0]	0.09
Successful passes [%]	65.3 ± 8.2	74.5 ± 6.6	< 0.01	[− 14.1, − 4.3]	1.23
Unsuccessful passes [%]	34.7 ± 8.2	25.5 ± 6.6	< 0.01	[3.8, 14.6]	1.06

## Discussion

The main findings of the study were that BB reduced distances covered in jogging, running and high-speed running compared to CA. Technical and tactical parameters showed an increased number of duels (p = 0.024; *d *= 0.89) and set pieces (p = 0.025; *d *= 1.00), while the mean time in ball possession per action (p = 0.021; *d *= 0.62) was reduced. The total number of passes was unaffected, but the rate of successful passes (p < 0.001; *d *= 1.24) was lower and the rate of unsuccessful passes (p < 0.001; *d *= 1.06) higher. There was a trend towards a lower difference in ball possession (*p *= 0.058; *d *= 0.69) between bio-banded teams.

Previous studies showed that BB reduces the dominance and physical advantage of early maturing players [1, 8], which have to strive to development better their technical and tactical skills to keep up with physically equal opponents [7]. Late maturing players, on the other hand, participate more actively in match play and perceive more options to show leadership [2, 4]. With higher self-confidence [4] and a reduced perception of injury risk [2], late maturing players are involved in more tackles [8].

The quicker change of match play situations between bio-banded teams in the present study was visible with the reduced time of ball possession per action and difference in ball possession between teams. The larger involvement of late maturing players and the reduced dominance of early maturing players in BB might result in a more technically and tactically challenging game. While the total number of passes remained unaffected, the number of unsuccessful passes was increased at the cost of successful passes. In addition, the number of duels and set pieces increased. The increase variation of match play situations between BB and conventional in the short term might however provide additional opportunities for skill acquisition over the long run [8, 16]. With greater involvement of late maturing players and higher technical and tactical challenges for early maturing players, a more variable match play between bio-banded teams might aid in the development of technical and tactical skills of young soccer talents [4].

In addition, the match play in BB seems to result in quicker ball movement. Previous studies showed a higher number of short passes, fewer dribbles and fewer long passes with BB [8]. The shift towards a quicker and more technically and tactically challenging game reduces, conversely, perceived exertion among late maturing players [8]. These findings are supported with the present study, showing reduced running distances among various submaximal velocities. While the reduced physical demand can be compensated with specific conditioning regimes, BB aids in developing specific on-ball actions, i.e. duels and set pieces.

## Conclusion

BB results in a quicker change of match play situations with a reduced difference in ball possession between teams and reduced time of ball possession per action. The increased number of duels, unsuccessful passes and set pieces seem to result from a quicker match play and higher involvement of late maturing players. While physical demand is reduced for the benefit of more technical and tactical match play, BB might support the development of technical and tactical skills in youth soccer.

## Limitations

While the nature of team sports involves the exchange of players during match play, future studies would benefit from a strict paired crossover design using the same positions in all matches. This would also enable analyses of the technical, tactical and physical parameters specific to playing position and maturity status. In addition, future studies need to evaluate the long-term effects, the influences of team interaction and the optimal time point and dose for its application of BB in youth soccer.

## Data Availability

All data are available on request.

## Abbreviations

APHV: Age at peak height velocity
BA: Biological age
BB: Bio-banding
CA: Chronological age
MO: Maturity offset
